# Multiscale Investigation of the Structural, Electrical and Photoluminescence Properties of MoS_2_ Obtained by MoO_3_ Sulfurization

**DOI:** 10.3390/nano12020182

**Published:** 2022-01-06

**Authors:** Salvatore E. Panasci, Antal Koos, Emanuela Schilirò, Salvatore Di Franco, Giuseppe Greco, Patrick Fiorenza, Fabrizio Roccaforte, Simonpietro Agnello, Marco Cannas, Franco M. Gelardi, Attila Sulyok, Miklos Nemeth, Béla Pécz, Filippo Giannazzo

**Affiliations:** 1 Consiglio Nazionale delle Ricerche—Istituto per la Microelettronica e Microsistemi (CNR-IMM), Strada VIII 5, 95121 Catania, Italy; SalvatoreEthan.Panasci@imm.cnr.it (S.E.P.); Emanuela.Schiliro@imm.cnr.it (E.S.); salvatore.difranco@imm.cnr.it (S.D.F.); giuseppe.greco@imm.cnr.it (G.G.); Patrick.Fiorenza@imm.cnr.it (P.F.); fabrizio.roccaforte@imm.cnr.it (F.R.); simonpietro.agnello@unipa.it (S.A.); 2 Department of Physics and Astronomy, University of Catania, 95123 Catania, Italy; 3 Centre for Energy Research, Institute of Technical Physics and Materials Science, Konkoly-Thege ut 29-33, 1121 Budapest, Hungary; koos.antal@ek-cer.hu (A.K.); sulyok.attila@ek-cer.hu (A.S.); nemeth.miklos@ek-cer.hu (M.N.); 4 Department of Physics and Chemistry Emilio Segrè, University of Palermo, 90123 Palermo, Italy; marco.cannas@unipa.it (M.C.); franco.gelardi@unipa.it (F.M.G.); 5 ATEN Center, University of Palermo, 90123 Palermo, Italy

**Keywords:** MoS_2_, sulfurization, XPS, Raman, TEM, C-AFM, photoluminescence

## Abstract

In this paper, we report a multiscale investigation of the compositional, morphological, structural, electrical, and optical emission properties of 2H-MoS_2_ obtained by sulfurization at 800 °C of very thin MoO_3_ films (with thickness ranging from ~2.8 nm to ~4.2 nm) on a SiO_2_/Si substrate. XPS analyses confirmed that the sulfurization was very effective in the reduction of the oxide to MoS_2,_ with only a small percentage of residual MoO_3_ present in the final film. High-resolution TEM/STEM analyses revealed the formation of few (i.e., 2–3 layers) of MoS_2_ nearly aligned with the SiO_2_ surface in the case of the thinnest (~2.8 nm) MoO_3_ film, whereas multilayers of MoS_2_ partially standing up with respect to the substrate were observed for the ~4.2 nm one. Such different configurations indicate the prevalence of different mechanisms (i.e., vapour-solid surface reaction or S diffusion within the film) as a function of the thickness. The uniform thickness distribution of the few-layer and multilayer MoS_2_ was confirmed by Raman mapping. Furthermore, the correlative plot of the characteristic A_1g_-E_2g_ Raman modes revealed a compressive strain (ε ≈ −0.78 ± 0.18%) and the coexistence of n- and p-type doped areas in the few-layer MoS_2_ on SiO_2_, where the p-type doping is probably due to the presence of residual MoO_3_. Nanoscale resolution current mapping by C-AFM showed local inhomogeneities in the conductivity of the few-layer MoS_2_, which are well correlated to the lateral changes in the strain detected by Raman. Finally, characteristic spectroscopic signatures of the defects/disorder in MoS_2_ films produced by sulfurization were identified by a comparative analysis of Raman and photoluminescence (PL) spectra with CVD grown MoS_2_ flakes.

## 1. Introduction

Transition metal dichalcogenides (TMDs) are a wide family of layered van der Waals (vdW) materials with the general chemical formula MX_2_, M being a transition metal (Ti, Zr, Hf, V, Nb, Ta, Mo, W, Re, Pd, or Pt) and X a chalcogen atom (S, Se, or Te) [1]. Most of them exhibit metallic or semiconducting phases. In particular, semiconducting TMDs have been the object of increasing scientific interest in the last decade, due to their huge potential for applications in several fields, including electronics, optoelectronics, spintronics, valleytronics, chemical/environmental sensing, energy generation, and catalysis [2,3,4,5,6,7,8,9,10]. Molybdenum disulfide (MoS_2_) is the most investigated among TMDs, due to the natural abundance and good chemical/mechanical stability of its 2H semiconductor phase under ambient conditions. The bandgap tunability as a function of the thickness, with a transition from an indirect bandgap of ~1.2 eV for bulk or few-layer MoS_2_ to a direct bandgap of ~1.8 eV for monolayer MoS_2_ [11,12], makes this material appealing for optoelectronic and electronic applications. In fact, the first robust 2D transistor with a large on/off ratio and good field-effect mobility was demonstrated using monolayer 2H-MoS_2_ flakes as the semiconducting channel [13,14]. This material and other TMDs are currently considered a potential replacement of Si for the next generation of complementary metal oxide semiconductor (CMOS) devices allowing the continuation of Moore’s law [15]. Furthermore, they can represent the basis for new concept (More-than-Moore) devices [16,17].

Due to this wide application potential, scalable and reproducible growth methods for thin films of TMDs are strongly required for their future implementation in manufacturing lines. In this context, research on MoS_2_ wafer-scale growth and device integration is relatively more mature than for other 2D TMDs.

Top-down synthesis approaches used to separate MoS_2_ from bulk crystals, such as mechanical exfoliation [18,19], gold-assisted exfoliation [20,21,22,23,24], and liquid exfoliation [25], are not suitable to ensure the reproducibility and thickness control on a wafer scale required for high-end electronic applications. For this reason, bottom-up approaches as Chemical Vapour Deposition (CVD) [26,27], Pulsed Laser Deposition (PLD) [28], Molecular Beam Epitaxy (MBE) [29], and Atomic Layer Deposition (ALD) [30] represent the most promising methods to obtain a reproducible thin film of TMDs on a large area.

In particular, CVD using vapours from S and MoO_3_ powders has been widely explored by several research groups, since it is a cost-effective method to produce MoS_2_ domains with good crystalline quality on different substrates [31,32,33]. Although monolayer flakes with a triangular or hexagonal shape and lateral extension from tens to hundreds of micrometres have been obtained under optimized CVD conditions [34], achieving coverage and thickness uniformity on the wafer scale still represents a huge challenge, due to the difficulty of controlling all the parameters involved in the process (including the substrate temperature, the evaporation rates of the S and Mo precursors, the pressure in the chamber, and the carrier gas flow rate) [35,36,37,38,39].

As an alternative to the single-step CVD approach, sulfurization of a Mo (or Mo-oxide) film pre-deposited on a substrate (e.g., by evaporation or sputtering) allows superior control of MoS_2_ coverage and uniformity by controlling the initial film thickness [40,41,42,43]. Different to CVD (where the Mo–S bonds are mostly formed by vapour phase reaction and the MoS_2_ lands on the substrate), the sulfurization process is a heterogeneous vapour-solid reaction between the S vapour and the pre-deposited film [44]. The conversion of MoOx to MoS_2_ by sulfurization has been demonstrated to occur in a wide temperature range, from 500 °C to 1000 °C, although the best quality films are typically obtained at temperatures > 750 °C [44]. Besides the vapour-solid surface reaction, the initial Mo or MoOx film thickness also plays an important role in the process. In fact, with increasing its thickness, the diffusion of S in the film represents the limiting mechanism for the formation of MoS_2_ layers and determines their alignment with respect to the substrate [45,46]. In particular, at typical sulfurization temperatures of 750–800 °C, single or few-layers of MoS_2_ horizontally aligned to the substrate plane are obtained for very thin (<3 nm) Mo films, whereas vertically aligned growth occurs for thicker Mo films [47]. This is due to the favoured sulphur diffusion along the vdW gaps between the vertically oriented MoS_2_ layers [45,47,48]. Besides the initial Mo (or Mo-oxide) thickness, other key factors controlling MoS_2_ formation include the substrate heating rate, pressure, and local S concentration on the sample surface [49,50,51]. Furthermore, the underlying substrate can play an important role in MoS_2_ formation during sulfurization of pre-deposited MoO_3_. In fact, while a higher temperature may enhance the sulfurization degree, on the other hand, it can also result in increased MoO_3_ evaporation and diffusion of Mo atoms on the substrate surface. This latter phenomenon strongly depends on the adhesion energy and surface diffusivity of Mo atoms on the substrate.

The main disadvantage of the continuous MoS_2_ films produced by the sulfurization approach is their nanocrystalline structure (with 20–30 nm grain-size) [44], typically resulting in poorer carrier mobility, if compared to the large and isolated monocrystalline MoS_2_ flakes obtained by the CVD approach. However, the high uniformity and its good compatibility with the fabrication methods used in the semiconductor industry makes this approach appealing for some applications, e.g., MoS_2_/semiconductor heterojunctions [52] or hydrogen evolution applications [53]. Hence, a detailed characterisation of structural/compositional, vibrational, optical, and electrical properties of MoS_2_ films produced by Mo sulfurization remains highly desirable.

In this paper, few or multilayer MoS_2_ on a SiO_2_/Si substrate have been produced by sulfurization at 800 °C of very thin MoO_3_ films, from ~2.8 nm to ~4.2 nm (i.e., the critical range for the transition from horizontally to vertically aligned layers). The compositional, morphological, structural, electrical, and optical emission properties of the grown films have been extensively investigated by the combination of several characterisation techniques with macro to nanoscale spatial resolution. This correlative analysis provides deep insight into the potentialities and limitations of this material system for applications.

## 2. Materials and Methods

**Figure 1:**
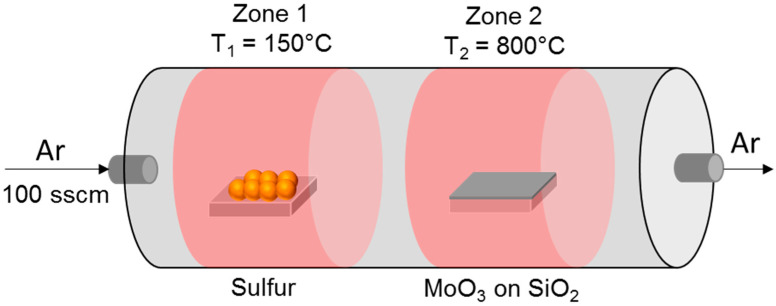
Schematic illustration of the sulfurization process of the thin MoO_3_ films on the SiO_2_/Si substrates.

The thin molybdenum-oxide films on SiO_2_ (900 nm)/Si substrates were obtained by DC magnetron sputtering from a Mo-target (using a Quorum Q300-TD system), followed by natural oxidation in air. The sulfurization process, schematically illustrated in Figure 1, was carried out in a two-heating zones furnace (TSH12/38/500, Elite Thermal Systems Ltd., Market Harborough, UK), with the first zone (at a temperature of 150 °C) hosting a crucible with 300 mg sulphur (purity 99.9%, product 28260.234, VWR Chemicals, Radnor, PA, USA), and the second zone (at a temperature of 800 °C) hosting the MoO_3_/SiO_2_/Si sample. Starting from a base pressure of 4 × 10^−6^ bar, the Ar carrier gas (purity 5.0, Messer, Budapest, Hungary) with a flux of 100 sccm transported the S vapours from the first to the second zone. The duration of the sulfurization process was 60 min.

Morphological analyses on the as-deposited MoO_3_ films and after the sulfurization process were carried out by Tapping mode Atomic Force Microscopy using a DI3100 system by Bruker (Santa Barbara, CA, USA) with Nanoscope V electronics. The compositional properties of the as-deposited metal films and MoS_2_ formation after the sulfurization process were evaluated by X-ray photoelectron spectroscopy (XPS) using Escalab Xi+ equipment by Thermo Fisher (Waltham, MA, USA), with a monochromatic Al Kα X-ray source (energy = 1486.6 eV). The spectra were collected at a take-off angle of 90° relative to the sample surface and pass energy of 20 eV. The instrument resolution was 0.45 eV (FWHM of the Ag 3d_5/2_ peak). The spectra were aligned using C1s (285 eV) as reference. High-resolution transmission electron microscopy (HR-TEM), high angle annular dark-field scanning transmission electron microscopy (HAADF-STEM), and energy dispersion spectroscopy (EDS) analyses of the MoS_2_ thin films were carried out with an aberration-corrected Titan Themis 200 microscope by Thermo Fisher (Waltham, MA USA). To this aim, cross-sectioned samples were prepared by a focused ion beam (FIB). Raman spectroscopy and mapping of MoS_2_ vibrational peaks were carried out by WiTec Alpha equipment by WiTec (Ulm, Germany), using laser excitation at 532 nm, 1.5 mW power, and 100× objective. Photoluminescence spectra (PL) were collected using a Horiba (Palaiseau, France) system with a laser source of 532 nm. To confirm the uniformity of the MoS_2_ thin layer across the substrate, the Raman and PL analyses have been performed at different positions on the sample. Finally, nanoscale resolution current mapping of MoS_2_ on SiO_2_ was performed by conductive Atomic Force Microscopy (C-AFM) with a DI3100 system by Bruker (Santa Barbara, CA, USA), using Pt-coated Si tips with ~5 nm curvature radius.

## 3. Results and Discussion

**Figure 2:**
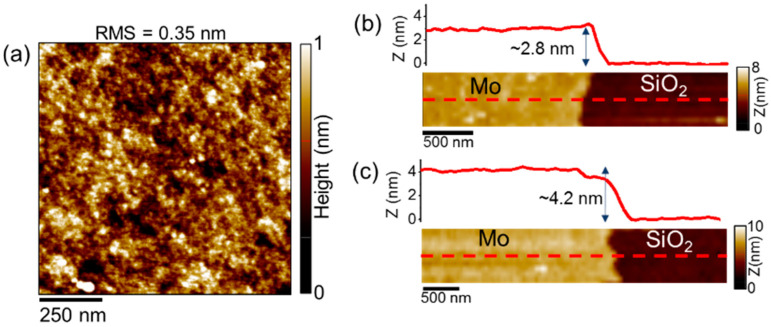
(**a**) Typical AFM morphology of as-deposited MoO_3_ thin films on SiO_2_, with the indication of the root mean square (RMS) roughness. (**b**,**c**) Determination of the thickness of films deposited with two different sputtering times by measurement of the step heights (~2.8 nm and ~4.2 nm) with respect to SiO_2_ on scratched regions.

Figure 2a shows a typical AFM morphology of as-deposited MoO_3_ on the SiO_2_/Si substrate using the lowest sputtering time (30 s). This analysis indicates a very low root mean square (RMS) surface roughness of 0.35 nm. Similar roughness values have been measured for MoO_3_ film thicknesses deposited at higher sputtering times. The thickness of the as-deposited films was also evaluated by AFM step height measurements performed on intentionally scratched regions of the films. Figure 2b,c show the morphologies and corresponding line profiles for films deposited with two different sputtering times (30 s and 45 s), resulting in ~2.8 nm and ~4.2 nm thickness, respectively.

XPS compositional analyses performed on the thinnest deposited films revealed that they are predominantly composed of MoO_3_, with a small (<1%) MoO_2_ contribution. Recently, Vangelista et al. [44] also reported the complete oxidation (ascribed to air exposure after the deposition) of evaporated Mo films with similar thickness, used for subsequent MoS_2_ growth by sulfurization. The same authors [44] explained the conversion of MoO_3_ to MoS_2_ upon exposure to sulphur according to the following chemical reaction:

2 MoO_3_(s) + 7 S(g) → 2 MoS_2_(s) + 3 SO_2_(g), (1)

which is the result of two intermediate steps:

MoO_3_ +(*x*/2) S → MoO_3−*x*_ + (*x*/2) SO_2_ (2)

MoO_3−*x*_ + [(7 − *x*)/2] S → MoS_2_ + [(3 − *x*)/2] SO_2_ (3)

i.e., the S-induced reduction of the MoO_3_ to a sub-stoichiometric oxide MoO_3−*x*_ (2), followed by its conversion to MoS_2_ (3), with the formation of gaseous SO_2_ as a by-product.

**Figure 3:**
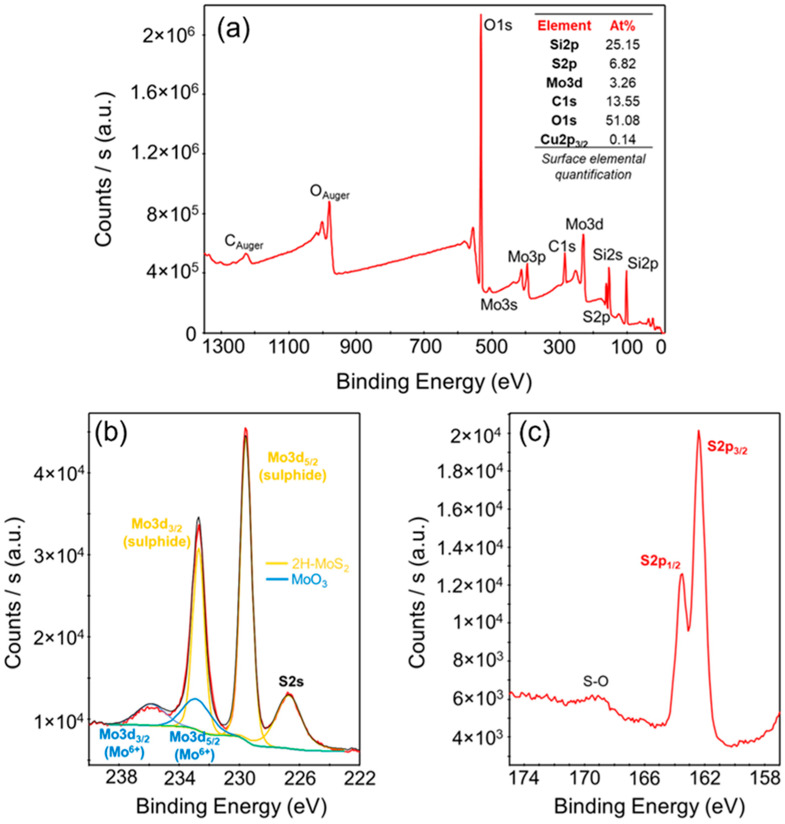
(**a**) Survey XPS spectrum of MoS_2_ on SiO_2_ produced by sulfurization of the 2.8 nm MoO_3_ film, with the indication of the evaluated surface elemental composition. (**b**) XPS spectra of the Mo 3d and S 2s core levels, with the deconvolution of the Mo^4+^ contribution (related to MoS_2_) and the Mo^6+^ contribution (related to residual MoO_3_). (**c**) S 2p core levels spectra, indicating the predominance of the sulphide contribution, with a small S-O component.

After the sulfurization process at 800 °C, XPS analyses were performed to evaluate the successful conversion of MoO_3_ to MoS_2_. Figure 3a reports an overview spectrum, allowing the quantification of the percentage of elemental concentrations on the sample surface. In particular, molybdenum and sulphur percentages of 3.26% and 6.82%, respectively, were evaluated (besides the large Si and O background), which were close to the stoichiometric [Mo]/[S] ratio for MoS_2._ More detailed information on the Mo and S bonding was deduced from the Mo3d_3/2_, Mo3d_5/2_, and S2s core levels in Figure 3b, and the S2p_1/2_ and S2p_3/2_ core levels in Figure 3c. Two doublets were found in the Mo 3d spectrum, and both doublets were fitted with a peak separation of 3.1 eV [44,54,55]. In particular, the deconvolution of the Mo3d peaks shows the predominance of the Mo^4+^ component, associated with 2H-MoS_2,_ accompanied by a smaller Mo^6+^ contribution, associated with the presence of residual MoO_3_. The two S2p_1/2_ and S2p_3/2_ peaks [44,54,55] in Figure 3c confirm that sulphur is mainly in the form of sulphide, with a small S-O component.

**Figure 4:**
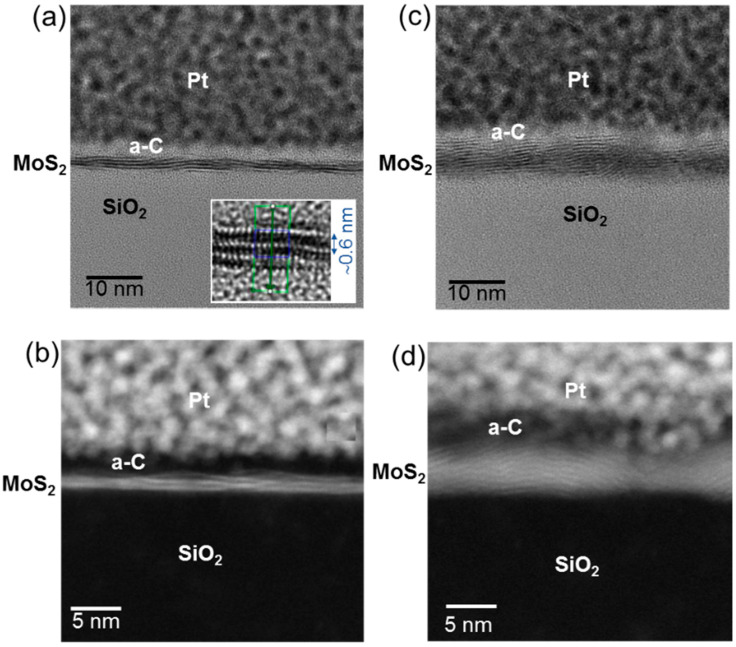
Cross sectional HR-TEM (**a**) and HAADF-STEM (**b**) images of few-layers MoS_2_ obtained by sulfurization of the ~2.8 nm MoO_3_ film on the SiO_2_ substrate. MoS_2_ is composed by nearly horizontally aligned 2–3 layers. The interlayer spacing in a 3-layers region is evaluated from the HR-TEM in the insert of panel (**a**). Cross sectional HR-TEM (**c**) and HAADF-STEM (**d**) of multilayers MoS_2_ obtained by sulfurization of the ~4.2 nm MoO_3_ film.

The structural properties of the MoS_2_ films were also investigated at nanoscale by transmission electron microscopy on cross-sectioned samples. Figure 4a,b show representative HR-TEM and HAADF-STEM analyses on the few-layers MoS_2_ sample obtained by sulfurization of the ~2.8 nm MoO_3_ film. The diffraction contrast in the HR-TEM image Figure 4a demonstrates the presence of two or three crystalline layers embedded between the amorphous SiO_2_ substrate and amorphous carbon (a–c) protective film. These layers are predominantly oriented parallel to the substrate, with nanometric scale corrugations. Furthermore, an interlayer spacing of ~0.6 nm is directly evaluated from the HRTEM image of a 3L-MoS_2_ reported in the insert of Figure 4a. The number of MoS_2_ layers and their nearly parallel orientation with respect to the substrate is confirmed by the HAADF-STEM image in Figure 4b collected on the same sample. On the other hand, a more irregular configuration of the layers can be observed from the HRTEM (Figure 4c) and HAADF-STEM (Figure 4d) analyses performed on the MoS_2_ multilayer produced by sulfurization of ~4.2 nm film. In fact, in the analysed specimen volume, horizontally oriented MoS_2_ layers co-exist with layers standing up with respect to the SiO_2_ surface. This observation is fully consistent with previous reports showing a transition from horizontal to vertically oriented growth for film thickness larger than 3 nm [47].

**Figure 5:**
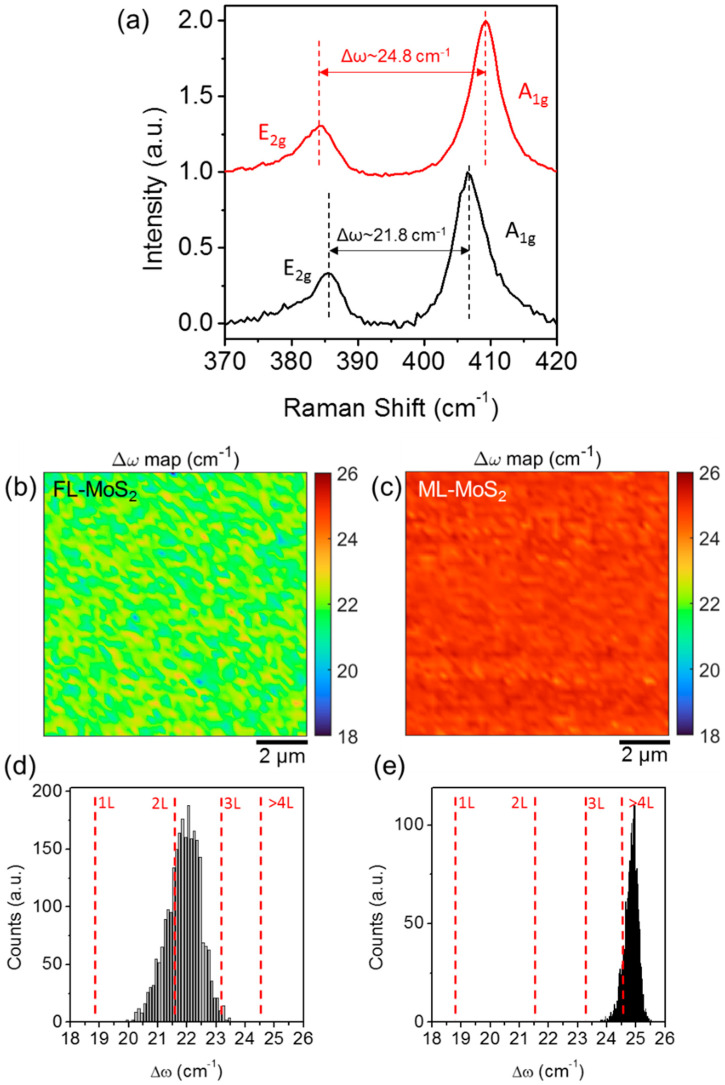
(**a**) Representative Raman spectra of the few-layers (FL) MoS_2_ (black-line) and multilayer (ML) MoS_2_ samples obtained by sulfurization of the 2.8 and 4.2 nm MoO_3_ films on SiO_2_. Colour maps of the A_1g_-E_2g_ wavenumber difference Δω obtained from arrays of Raman spectra collected on 10 μm × 10 μm scan areas on the FL-MoS_2_ (**b**) and on the ML-MoS_2_ (**c**) samples. Histogram of Δω values showing a distribution with a peak at ω ≈ 21.8 ± 0.6 cm^−1^ for the FL-MoS_2_ sample associated to 2 L–3 L MoS_2_ (**d**) and ω ≈ 24.8 ± 0.4 cm^−1^ for the ML-MoS_2_ sample, corresponding to >4 L MoS_2_ thickness (**e**).

The layers number uniformity of the grown MoS_2_ films was also investigated on micro-meter scale areas and with high statistics by Raman spectroscopy. Figure 5 shows two typical Raman spectra of the few-layers (i.e., 2 L–3 L) MoS_2_ (black line) and of the multilayer MoS_2_ (red line) grown on SiO_2_ by the sulfurization process. The two characteristic in-plane (E_2g_) and out-of-plane (A_1g_) vibrational modes of MoS_2_ are clearly identified, and the typical redshift of the E_2g_ peak and blue shift of the A_1g_ with increasing the number of layers [19] is observed. In particular, the difference $\Delta \omega =\omega_{A1g}-\omega_{E2g}$ between the wavenumbers of these two main modes is commonly taken as a way to evaluate the number of MoS_2_ layers, with larger Δω values generally associated with a thicker MoS_2_.

The colour maps in Figure 5b,c illustrate the spatial distribution of the Δω values obtained from arrays of 50 × 50 Raman spectra collected on 10 μm × 10 μm scan areas. Figure 5d,e show the histograms of the Δω values reported in the two maps, with the indication of the corresponding number of MoS_2_ layers according to the calibration reported in Ref. [19]. The two distributions are quite uniform and exhibit a ω ≈ 21.8 ± 0.6 cm^−1^ for the few-layer MoS_2_ sample and ω ≈ 24.8 ± 0.4 cm^−1^ for the multilayer MoS_2_ sample. These Δω values are associated with a 2 L–3 L MoS_2_ thickness for the first sample, in very good agreement with TEM analyses in Figure 4, and to >4 L MoS_2_ for the second one.

In the following, we will concentrate our attention on the 2 L–3 L MoS_2_ sample, since the horizontal configuration of the layers makes it more suitable for electronic applications, similarly to 2H-MoS_2_ samples produced by CVD or by exfoliation from bulk molybdenite.

**Figure 6:**
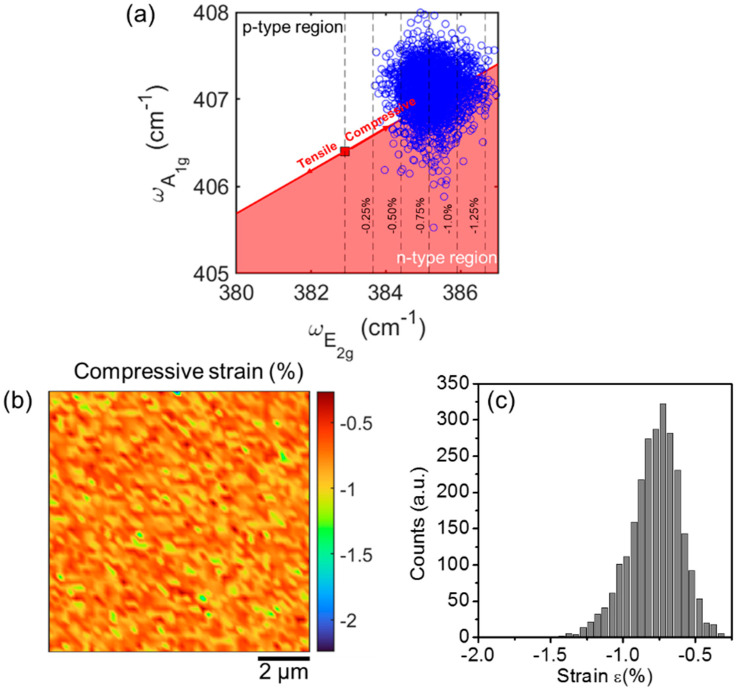
(**a**) Correlative plot of the ω_A1g_ and ω_E2g_ values (blue circles) extracted from all the Raman spectra in the array of Figure 5. The red line represents the ideal ω_A1g_ vs. ω_E2g_ dependence (i.e., the strain line) for a purely strained 3L-MoS_2_ film. The red square corresponds to the frequencies $\omega_{E_{2g}^{0}}$ and $\omega_{A_{1g}^{0}}$ for an ideally unstrained and undoped 3L-MoS_2_, while the two red arrows with opposite directions along the strain line indicate the tensile (red-shift) and compressive strain (blue-shift), respectively. (**b**) Map and (**c**) corresponding histogram of the compressive strain on a 10 μm × 10 μm area.

The doping type and the biaxial strain (ε) of the thin MoS_2_ film were also evaluated from the Raman maps by a correlative plot of A_1g_ versus E_2g_ peaks positions, as recently discussed in Ref. [23]. Figure 6a shows as blue circles the $\omega_{A1g}$ and $\omega_{E2g}$ values extracted from all the Raman spectra in the array of Figure 5. The red line in Figure 6a represents the ideal $\omega_{A1g}\;\mathrm{vs}.{\;\omega }_{E2g}$ dependence (i.e., the strain line) for a purely strained 3L-MoS_2_ film. This relation is obtained from the combination of the following two expressions:

(4) $$\omega_{E_{2g}}=\omega^{0}{}_{E_{2g}}-2\gamma_{E_{2g}}\omega^{0}{}_{E_{2g}}\varepsilon$$

(5) $$\omega_{A_{1g}}=\omega^{0}{}_{A_{1g}}-2\gamma_{A_{1g}}\omega^{0}{}_{A_{1g}}\varepsilon$$

Here, $\gamma_{E_{2g}}=0.39$ and $\gamma_{A_{1g}}=0.09$ are the Grüneisen parameters for the two vibrational modes of 3L-MoS_2_, estimated from the literature values of the peaks shift rates as a function of strain percentage (−3 cm^−1^/% and −0.7 cm^−1^/% for the E_2g_ and A_1g_ peaks, respectively) [56]. $\omega_{E_{2g}^{0}}$ and $\omega_{A_{1g}^{0}}$ represent the E_2g_ and A_1g_ frequencies for an ideally unstrained and undoped 3L-MoS_2_. Here, the literature values for a suspended 3L-MoS_2_ membrane ($\omega^{0}{}_{E_{2g}}=382.9{\;\mathrm{cm}}^{-1}$ and $\omega^{0}{}_{A_{1g}}=406.4{\;\mathrm{cm}}^{-1}$) [56], not affected by the interaction with the substrate, were taken as the best approximation for these ideal values. This reference point is reported as a red square in Figure 6a, while the two arrows with opposite directions along the strain line indicate the tensile (red-shift) and compressive strain (blue-shift), respectively. Furthermore, the black dashed lines serve as guides to estimate the strain values. The distribution of the experimental points (blue circles) in the plot of Figure 6a clearly indicates that the thin MoS_2_ film on SiO_2_ is compressively strained. Figure 6b shows the 2D map of the compressive strain, calculated from the map of $\omega_{E2g}$ values by applying Equation (4). Furthermore, the corresponding histogram of the ε values is reported in Figure 6c, from which an average strain value ε≈−0.78%±0.18% can be deduced.

The strain line separates the n-type and p-type doping regions in the $\omega_{A1g}$ − $\omega_{E2g}$ diagram in Figure 6a. Noteworthy, the experimental points in Figure 6a are partially located in the n-type region and partially in the p-type one. Unintentional n-type doping is typically reported for MoS_2_ films produced by different synthesis methods (such as mechanical exfoliation or CVD) and it is commonly ascribed to native defects present in the material [57,58,59,60]. Here, the observed p-type doping in some regions of the MoS_2_ film produced by sulfurization can be associated with the presence of residual MoO_3_, as deduced by XPS. In fact, several studies demonstrated how intentionally introducing MoO_3_ in pristine (n-type) MoS_2_, e.g., by O_2_ plasma treatments, results in p-type doping of the material [61,62].

**Figure 7:**
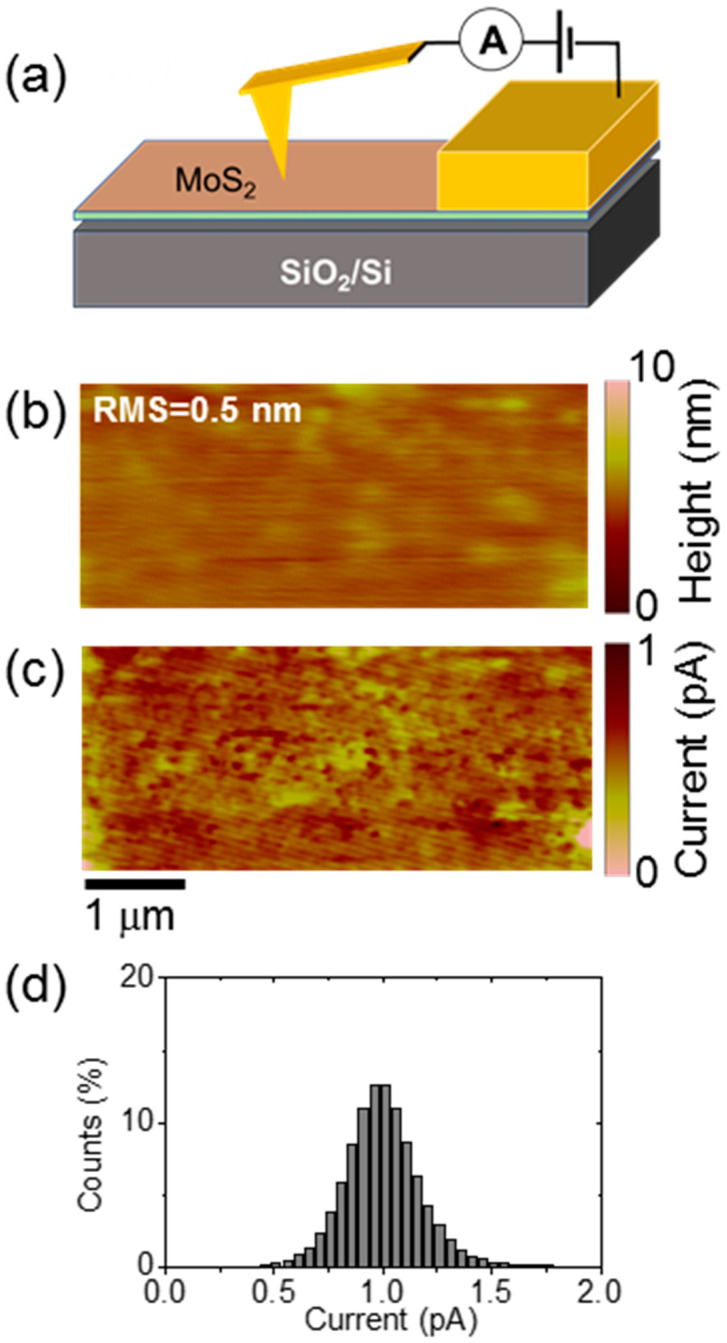
(**a**) Schematic of the C-AFM setup for local conductivity mapping of few-layers MoS_2_ on SiO_2_. (**b**) Morphology and (**c**) current map simultaneously measured with tip-to-sample bias of 5 V. (**d**) Histogram of current values from the C-AFM map.

The MoS_2_ thin layers produced by MoO_3_ thin films sulfurization exhibit large resistivity values in the range of 10–100 Ω∙cm [63]. This can be ascribed, in part, to the nanocrystalline structure of the films, i.e., the large density of grain boundaries, which are known to introduce resistive contributions in the current path [64]. On the other hand, the local changes in the compressive strain distribution, as well in the carrier density, deduced by Raman mapping is expected to have an effect on the electrical properties of the few-layers of MoS_2_. To get direct information on the homogeneity of conductivity in this film, local current mapping has been carried out by C-AFM, as schematically depicted in Figure 7a. In this configuration, the current locally injected from the AFM metal tip flows in the MoS_2_ film and is finally collected from the macroscopic front contact. Due to the nanoscale size of the tip contact, the dominant contributions to the measured resistance are represented by the local tip/MoS_2_ contact resistance and the spreading resistance in the MoS_2_ region underneath the tip. Figure 7b shows the contact-mode morphological image on the sample surface, from which an RMS roughness ≈ 0.5 nm slightly higher than the one of the as-deposited MoO_3_ film (Figure 2a) was deduced. Figure 7c,d report the corresponding C-AFM current map and the histogram of the measured current values. The current map clearly shows submicrometer lateral variations of the conductivity, which are only partially correlated to the morphology, while the histogram shows a Gaussian distribution of these values, resembling the shape of the strain distribution in Figure 7d. From this comparison, we can speculate that these mesoscopic-scale inhomogeneities can be partially ascribed to the lateral changes in the strain and carrier density detected by Raman.

In the last section of this paper, Raman and photoluminescence spectra acquired on the few-layers MoS_2_ samples produced by sulfurization have been compared with reference spectra acquired on CVD-grown MoS_2_ samples with a similar thickness.

**Figure 8:**
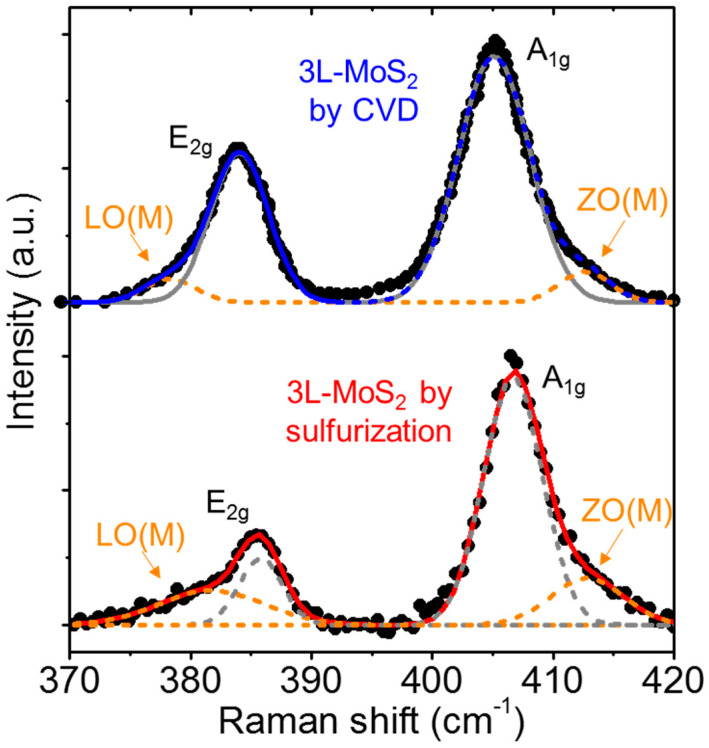
Raman spectrum for 3L-MoS_2_ produced by sulfurization (red), compared with a reference spectrum for CVD grown 3L-MoS_2_ (blue). Data for CVD 3L-MoS_2_ were adapted with permission from [65], copyright Elsevier 2020.

Figure 8 shows a typical Raman spectrum of 3L-MoS_2_ on SiO_2_ produced by MoO_3_ sulfurization, compared with a spectrum of a 3L-MoS_2_ sample grown by CVD on SiO_2_ [65], reported as reference. Some remarkable differences can be clearly observed between MoS_2_ layers prepared using the two different approaches. In fact, besides a lower E_2g_/A_1g_ intensity ratio, the two vibrational peaks exhibit a more pronounced asymmetric shape in the 3L-MoS_2_ produced by sulfurization as compared to the CVD-grown one. The deconvolution analysis of the Raman spectra with four Gaussian contributions, associated with the main E_2g_ and A_1g_ modes and the disorder activated LO(M) and ZO(M) modes [66], is also presented in Figure 8. These LO(M) and ZO(M) components are very small in the Raman spectra of CVD 3L-MoS_2_, whereas their weight is higher in the 3L-MoS_2_ produced by sulfurization. In this latter case, they can be ascribed both to the nanocrystalline nature of the film, as well as to the presence of residual MoO_3_, as deduced from the XPS analyses.

**Figure 9:**
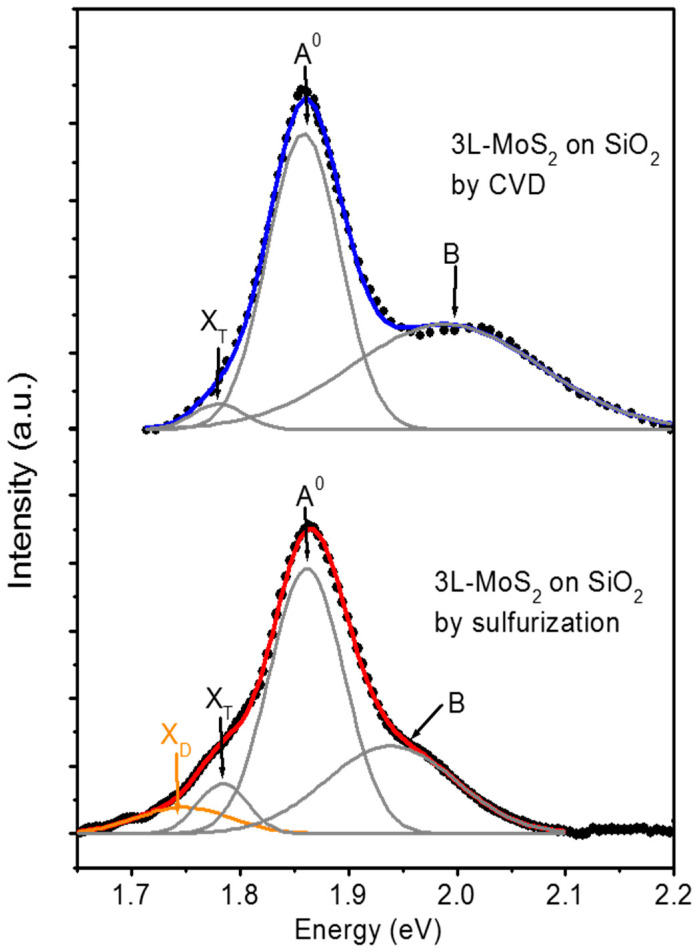
Photoluminescence (PL) spectra for 3L-MoS_2_ produced by sulfurization, compared with a reference spectrum for CVD grown 3L-MoS_2_. The deconvolution analysis indicated the presence of the excitonic contributions A^0^, B, and of the trionic contribution X_T_ (grey lines) for the CVD grown sample. In addition, the defect-related peak X_D_ (orange line) is identified in the sulfurization grown sample. Data for CVD 3L-MoS_2_ were adapted with permission from [65], copyright Elsevier 2020.

Figure 9 shows the comparison between a PL spectrum measured on the 3L-MoS_2_ produced by sulfurization with a reference spectrum for CVD grown 3L-MoS_2_, taken from Ref. [65]. For both spectra, acquired using a 532 nm wavelength laser source, the main emission peak at an energy of 1.86 eV can be observed. However, significant differences in spectral features can be clearly identified from a detailed deconvolution analysis.

The PL spectrum of CVD MoS_2_ can be fitted by three Gaussian peaks, associated with the two exciton contributions (A^0^ at 1.86 ± 0.01 eV and B at 1.99 ± 0.01 eV, due to the spin-orbit splitting of the valence band) and the trionic contribution (X_T_ at 1.78 ± 0.01 eV) [65]. On the other hand, the deconvolution analysis of the spectrum for the sulfurization grown sample allowed us to identify a fourth component X_D_ at 1.75 ± 0.01 eV, besides the trion (X_T_ at 1.78 ± 0.01 eV) and exciton peaks (A^0^ at 1.86 ± 0.01 eV and B at 1.95 ± 0.01 eV). Noteworthy, the presence of this X_D_ contribution is accompanied by a strong decrease in the spectral weight of the exciton peak B, as compared to the case of the CVD sample, as well as its FWHM reduction. The occurrence of a similar feature X_D,_ associated with point defects in the MoS_2_ lattice, has been recently reported by Chow et al. [67] for the PL spectra of MoS_2_ flakes subjected to soft Ar-plasma irradiation, and it was also accompanied by a decrease in the exciton peak B with respect to unirradiated flakes. Hence, the observed X_D_ contribution for our samples produced by sulfurization was ascribed to a higher density of point defects with respect to CVD grown samples.

## 4. Conclusions

In conclusion, we reported a detailed analysis of the compositional, morphological, structural, electrical, and optical emission properties of few or multilayer MoS_2_ on a SiO_2_/Si substrate produced by sulfurization of very thin MoO_3_ films at 800 °C. Both Raman mapping and TEM/STEM analyses showed the formation of 2–3 layers of MoS_2_ nearly aligned with the SiO_2_ surface after sulfurization of the thinnest MoO_3_ film, whereas multilayers of MoS_2_ (partially standing up) were observed for the thicker MoO_3_ film. The strain distribution in the few-layer MoS_2_ on SiO_2_ was evaluated by the correlative plot of the characteristic A_1g_-E_2g_ Raman modes, showing the occurrence of a compressive strain ε ≈ −0.78 ± 0.18%. Furthermore, the co-existence of submicrometer areas with n- and p-type doping is detected, with the p-type doping probably due to the presence of residual MoO_3_, as revealed by XPS analyses. Nanoscale resolution current mapping by C-AFM showed conductivity inhomogeneities in the few-layer MoS_2_, which are well correlated to the lateral changes in the strain detected by Raman. Finally, the characteristics spectroscopic signatures of the defects/disorder were identified by comparing Raman and PL spectra of sulfurization grown MoS_2_ with reference analyses of CVD-grown single crystalline MoS_2_.

The demonstrated MoS_2_ growth method is quite versatile and can be extended to different substrates, besides SiO_2_. In particular, the adoption of crystalline substrates (such as sapphire, GaN, and 4H-SiC) with the hexagonal basal plane and good lattice matching with MoS_2_ is expected to enhance the domain size and electronic quality of the grown films. Furthermore, the homogeneous large area few-layer MoS_2_ can be transferred to arbitrary substrates (including flexible ones) [68] and find applications in different fields of microelectronics, flexible electronics, and sensing.

## Data Availability

The data that support the findings of this study are available from the corresponding author upon reasonable request.
